# Integrated analyses of DNA methylation and hydroxymethylation reveal tumor suppressive roles of *ECM1*, *ATF5*, and *EOMES* in human hepatocellular carcinoma

**DOI:** 10.1186/s13059-014-0533-9

**Published:** 2014-12-03

**Authors:** Fei Gao, Yudong Xia, Junwen Wang, Zhilong Lin, Ying Ou, Xing Liu, Weilong Liu, Boping Zhou, Huijuan Luo, Baojin Zhou, Bo Wen, Xiuqing Zhang, Jian Huang

**Affiliations:** BGI-Shenzhen, Shenzhen, 518083 China; National Engineering Center for Biochip at Shanghai, Shanghai, 201203 China; Shanghai-MOST Key Laboratory for Disease and Health Genomics, Chinese National Human Genome Center at Shanghai, Shanghai, 201203 China; Shenzhen Key Lab. of Infection and Immunity, Shenzhen Third People’s Hospital, Guangdong Medical College, Shenzhen, 518112 China; Guangdong Key Lab. Of Diagnosis & Treatment for Emerging Infectious Disease, Shenzhen Third People’s Hospital, Guangdong Medical college, Shenzhen, 518112 China

## Abstract

**Background:**

Differences in 5-hydroxymethylcytosine, 5hmC, distributions may complicate previous observations of abnormal cytosine methylation statuses that are used for the identification of new tumor suppressor gene candidates that are relevant to human hepatocarcinogenesis. The simultaneous detection of 5-methylcytosine and 5-hydroxymethylcytosine is likely to stimulate the discovery of aberrantly methylated genes with increased accuracy in human hepatocellular carcinoma.

**Results:**

Here, we performed ultra-performance liquid chromatography/tandem mass spectrometry and single-base high-throughput sequencing, Hydroxymethylation and Methylation Sensitive Tag sequencing, HMST-seq, to synchronously measure these two modifications in human hepatocellular carcinoma samples. After identification of differentially methylated and hydroxymethylated genes in human hepatocellular carcinoma, we integrate DNA copy-number alterations, as determined using array-based comparative genomic hybridization data, with gene expression to identify genes that are potentially silenced by promoter hypermethylation.

**Conclusions:**

We report a high enrichment of genes with epigenetic aberrations in cancer signaling pathways. Six genes were selected as tumor suppressor gene candidates, among which, *ECM1*, *ATF5* and *EOMES* are confirmed via siRNA experiments to have potential anti-cancer functions.

**Electronic supplementary material:**

The online version of this article (doi:10.1186/s13059-014-0533-9) contains supplementary material, which is available to authorized users.

## Background

Hepatocellular carcinoma (HCC), which is frequently caused by hepatitis virus (B and C) infection and alcohol abuse, is the most common type of primary liver cancer and third leading cause of cancer death worldwide [1,2]. Although surgical and chemotherapeutic treatment of HCC is evolving, surgical resection remains the treatment of choice for many patients. Surgical resection for HCC patients is associated with a 5-year survival rate of 50%; however, there is a 70% recurrence rate [3].

The mechanism underlying HCC development remains poorly understood. It is widely accepted that accumulating genetic alterations such as chromosomal alterations, gene amplifications, and mutations are associated with HCC [4,5]. Furthermore, epigenetic alterations, particularly abnormal DNA methylation at the 5 position of cytosine (5mC), have been extensively studied [6]. DNA hypomethylation in cancer cells is thought to lead to chromosomal instability and oncogene activation [7] and has generally been regarded as a highly stable clinical marker for cancer [8]. More importantly, a number of studies have reported that the hypermethylation of tumor suppressor genes (TSGs) contributes to HCC pathogenesis [9-11]. Thus, the accurate detection of DNA methylation may provide powerful mechanistic insight into hepatocarcinogenesis and may have a potential application for the clinical diagnosis of HCC.

However, the differences in 5-hydroxymethylcytosine (5hmC) distributions may complicate previous observations regarding abnormal cytosine methylation status. Previous technologies, such as bisulfite treatment and restriction enzyme-based technologies, are unable to distinguish between 5mC and 5hmC [12,13], and the existence of 5hmC in samples reduces the accuracy of DNA methylation detection [14]. 5hmC is catalyzed by ten-eleven translocation (*TET*) proteins, which first convert 5mC to 5hmC, then to 5-formylcytosine (5fC) and finally to 5-carboxylcytosine (5caC), thereby may play a role in DNA demethylation [15,16]. 5hmC was found to be abundant in embryonic stem cells and neurons, but it is greatly reduced in tumor cells [15,17-20], including HCC cells [21].

Therefore, there is a renewed interest for the simultaneous detection of 5mC and 5hmC in the context of genomic profiling studies, which may stimulate the discovery of aberrantly methylated genes with increased accuracy in HCC cells. Till now, the number of known aberrantly promoter-methylated genes is fewer for HCC than for colon and gastric cancer [5,6]. Furthermore, a more comprehensive study of the 5hmC status in HCC is required to determine its role in hepatocarcinogenesis.

To fully examine 5mC and 5hmC status in HCC, we used ultra-performance liquid chromatography/tandem mass spectrometry (UPLC-MS/MS) and a newly developed single-base high-throughput sequencing approach (hydroxymethylation and methylation sensitive tag sequencing (HMST-seq)) to synchronously measure these two modifications in HCC samples and their adjacent non-cancerous liver tissues (non-HCCs). We report a global loss of 5hmC and key genes containing altered methylation or hydroxymethylation that are enriched for important cancer-relevant signaling pathways. In particular, we identified three new genes (*ECM1*, *ATF5*, and *EOMES*) with potential anti-cancer functions that may promote the understanding of the molecular mechanisms of HCC development and progression and potentiate the future clinical applications of 5hmC detection.

## Results

### Globally increased 5mC but decreased 5hmC levels at genomic CCGG loci in HCC

We first performed UPLC-MS/MS to investigate global 5mC and 5hmC levels in 16 pairs of HCC and non-HCC samples and two HCC cell lines (97 L and LM6 cells). We found that both 5mC and 5hmC were frequently decreased in 71% and 100% of the HCC specimens, respectively, compared with non-HCC specimens. Correspondingly, 5mC and 5hmC levels in the two HCC cell lines remained relatively low (Additional file 1: Tables S2). These results are in accordance with previous reports of a global loss of 5mC and 5hmC in HCC and other cancer types [18,20,21].

To further examine the distribution of 5mC and 5hmC in HCC specimens and cell lines, we applied a newly developed technology, ‘HMST-seq’ [22], on seven of the 16 pairs of HCC specimens and the two cell lines. HMST-seq can detect 5mC and 5hmC at ‘CCGG’ sites with a single-base resolution, in which three different libraries were constructed for each sample including a library containing unmodified C, mC, and hmC tags through *Msp*I-mediated digestion of normal genomic DNA, a library containing C and mC tags through *Msp*I-mediated digestion of glucosylated genomic DNA and a library containing only C tags through *Hpa*II-mediated digestion of normal genomic DNA [22]. During manual examination of the tag distribution of these three libraries, we filtered out samples with similar distributions of ‘C + mC’ tags and ‘C’ tags, which indicate potential insufficient *Msp*I-mediated digestion of glucocylated genomic DNA (Additional file 2: Figure S1). As a result, three out of the seven matched pairs of HCC and non-HCC specimens passed our quality control. We then used these three matched pairs of specimens to infer methylation and hydroxymethylation status across all CCGG sites. On average, 170 million reads for each library were generated, and 147 million (86.58%) reads were aligned to a virtual library of the human genome (see the Methods section). An average of 100 M (58.93%) uniquely aligned reads were then obtained for each library, resulting in a minimum of 1.3 M informative ‘CCGG’ sites with greater than 50 X sequencing depth for each sample (Additional file 1: Table S3). Data normalization for the three libraries was performed, and significantly modified sites with 5mC or 5hmC were identified with our established methods (sequencing depth >10 X, FDR <0.001) [22]. On average, 433,045 (31.49%) and 72,080 (5.24%) total examined CCGG sites were identified as significant for 5mC and 5hmC, respectively, in non-HCC specimens. In addition, increased 5mC (471,270; 34.72%) but decreased 5hmC (66,020; 4.89%) was observed in the HCC specimens (Additional file 1: Table S2).

**Figure 1:**
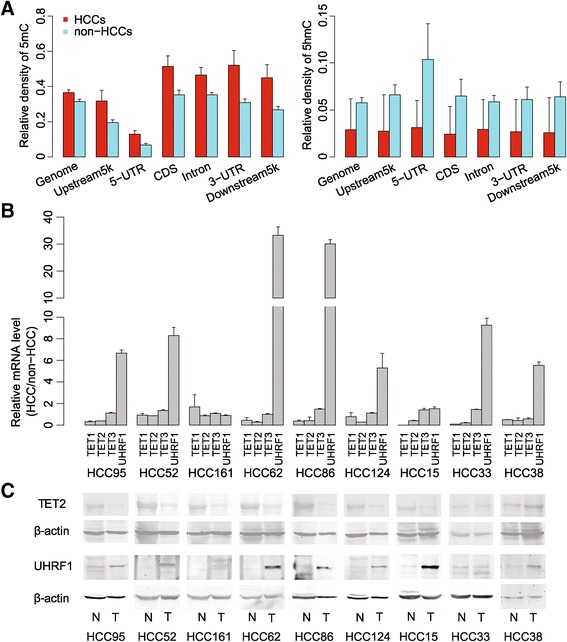
**The relative density of 5mC and 5hmC within genomic regions. (A)** The relative density indicates the ratio between 5mC or 5hmC and all inspected ‘CCGG’ sites within each genomic region. **(B)** The relative mRNA levels of UHRF1, TET1, 2, and 3 in HCCs and adjacent non-HCC liver tissues tested by RT-qPCR. **(C)** The expression of TET2 and UHRF1 protein in HCCs and adjacent non-HCC liver tissues using western blot. β-actin was used as loading control.

Based on these data, a broad distribution of 5mC and 5hmC was observed (Figure 1). Compared with the background distribution of all ‘CCGG’ sites in the genome, 5mC was more deficient in regulatory regions such as enhancers or regions surrounding transcriptional start sites (TSS) but relatively enriched in exons and intragenic regions. In contrast, the distribution of 5hmC was more evenly distributed with a background distribution across all ‘CCGG’ sites (Additional file 2: Figure S2). In agreement with previous reports that CpG islands are hypermethylated in tumors [8], globally increased 5mC was observed in CCGG sites across the genome of HCC compared with non-HCC samples (Figure 1A). In contrast, 5hmC levels of CCGG sites across the HCC genome were decreased, in consistence with whole-genome 5hmC decrease tested by UPLC-MS/MS.

As TET proteins are responsible for oxidizing 5mC to 5hmC [15,16], we tested mRNA levels of *TET1*, *2*, and *3* in nine pairs of HCC specimens. We found downregulation of mRNA levels of *TET1* and *TET2*, but not *TET3*, in HCCs in comparison with non-HCC samples (Figure 1B). Consistent with its mRNA level, TET2 protein was confirmed downregulated in HCCs as well (Figure 1C). We also tested UHRF1 as its protein helps to recruit DNMT1 to hemi-methylated DNA to facilitate faithful maintenance of DNA methylation [23]. In contrast to TET genes, both mRNA and protein expression levels of *UHRF1* gene were highly upregulated in HCCs (Figure 1B and C), consistent with previous reports in various types of cancers [24]. Based on these results, expression level changes of key enzymes or cofactors that are involved in DNA methylation/hydroxymethylation regulation might be correlated with 5mC increase and 5hmC decrease in CCGG sites across the HCC genome.

### DMRs and DhMRs are revealed by inter-group comparisons

**Figure 2:**
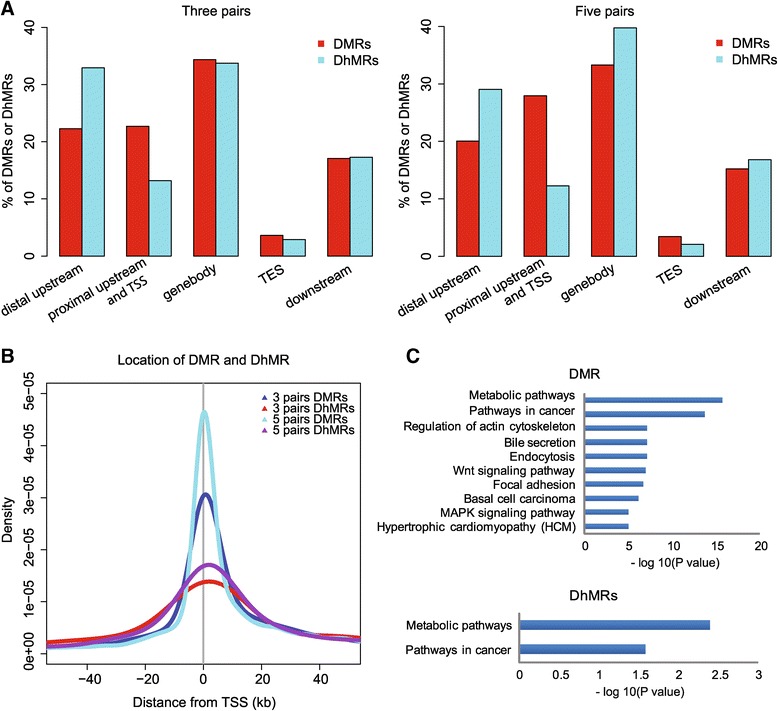
**The distribution of DMRs and DhMRs. (A)** The distribution of DMRs and DhMRs within genomic regions. The DMRs and DhMRs, located in two different elements, are attributed to regions that overlap with a bigger proportion of the DMR or DhMR and counted only once. **(B)** The distance between DMRs and DhMRs and transcriptional start sites (TSSs). The densities of DMRs and DhMRs with different distances from TSSs are displayed throughout regions of 40 kb upstream and downstream from TSSs. **(C)** KEGG pathway enrichment analysis of genes related to DMRs or DhMRs. The *x*-axis shows the *P* value from hypergeometric test adjusted by the multiple test adjustment.

Based on the observation of global methylation and hydroxymethylation alterations, we next aimed to specify differentially methylated regions (DMRs) and differentially hydroxymethylated regions (DhMRs) between HCC and non-HCC samples. A sliding window strategy was used for inter-group comparison of the HCC and non-HCC groups as previously described [22]. To identify differentially modified regions containing at least five CCGG sites (Wilcoxon rank-sum test, *P* <0.05), we began with the three matched pairs and found 1,851 DMRs and 243 DhMRs (Additional file 1: Table S4, S5). Although both modifications were widely distributed across the genome, DMRs occurred more frequently at proximal regions close to TSSs, while DhMRs were more likely to occur at distal sites upstream of TSSs (Figure 2A and B). Thus, only 10 (4.12%) DhMRs overlapped with DMRs, suggesting that changes in these two modifications rarely co-occur.

Furthermore, we compared the 5mC and 5hmC levels of the DMRs and DhMRs, respectively, among HCC and non-HCC samples and two HCC cell lines (97 L and LM6). 97 L and LM6 were derived from the same HCC patient but exhibit highly different metastatic potentials [25]. Similar to the HCC samples, hypermethylation of the DMRs and hypohydroxymethylation of the DhMRs were observed in 97 L and LM6 compared with non-HCC cells. Notably, the LM6 cells, which have a higher metastatic potential, exhibited a slightly higher methylation level in the DMRs compared with 97 L cells (Additional file 2: Figure S3).

To replicate above results, we re-performed HMST-Seq library construction on two pairs of the samples that first failed our quality control. Similar data quality and 5mC and 5hmC levels were achieved (Additional file 1: Table S2, S3). By combining all qualified data, an inter-group comparison of five matched pairs of HCC and non-HCC samples was re-performed, resulting in a total of 5,126 DMRs and 571 DhMRs using the same window-sliding strategy (Additional file 1: Table S4, S5); similar results regarding the distribution of DMR and DhMR were observed (Figure 2A, B).

### Enrichment of genes containing DMRs and DhMRs in cancer-related pathways

To further address the functional effects of DNA modification changes in HCC, we first performed KEGG pathway enrichment analysis (see the Methods section) of 1,373 DMR and 218 DhMR genes from the three matched pairs of HCC and non-HCC samples, respectively. A higher enrichment rate for DMR (16.8%; 231 out of 1373) than DhMR genes (8.7%, 19 out 218; FDR <0.05) was found (Additional file 1: Table S6). Although DMR and DhMR genes were enriched in KEGG cancer and metabolic pathways (Figure 2C), these genes rarely overlapped (Additional file 2: Figure S4). In addition, enrichment analysis of 3,283 DMR and 651 DhMR genes from the five pairs of HCC and non-HCC samples, respectively, revealed similarly enriched pathways (data not shown). Notably, cancer-related pathways including ‘Pathways in cancer’, ‘Focal adhesion’, ‘Wnt signaling pathway’, and ‘MAPK signaling pathway’ were enriched for DMR genes.

**Figure 3:**
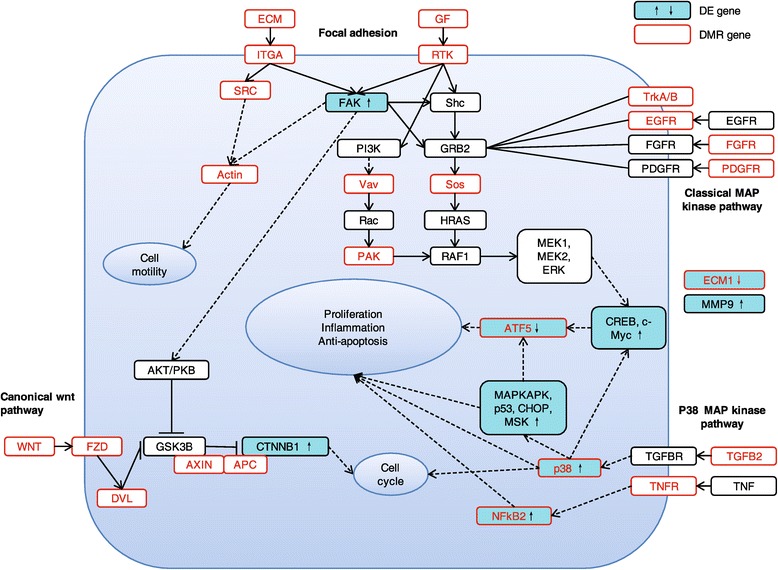
**Genes in cancer-related pathways.** Genes in the ‘Focal adhesion’, ‘Wnt signaling pathway’ and ‘MAPK signaling’ pathway. The cyan rectangle indicates differentially expressed genes (DE genes), and the red rectangle indicates genes with a differentially methylated region (DMR gene).

Focal adhesion components are crucial for the cell growth, movement, differentiation, and tailoring of the extracellular microenvironment [26], and of these genes, focal-adhesion kinase (*FAK*) is an important mediator involved in cancer formation and progression [27]. Multiple genes that encode extracellular proteins and transmit signals, including extracellular matrix (ECM), integrin, SRC, and growth factor (GF) genes, were hypermethylated in HCCs. Signaling by the Wnt family of secreted glycolipoproteins via the transcriptional co-activator β-catenin can also determine cell fate and has been implicated in cancer proliferation and survival, including HCC cells [28]. Aberrant methylation was also observed for key genes of the Wnt signaling pathway including the *Wnt*, *FZD*, and *APC* genes. Furthermore, multiple key genes in the MAPK signaling pathway were enriched among those containing DMRs, including *TGFB2*, *EGFR*, *NFkB2*, and *p38* mitogen-activated protein kinase (Figure 3, Additional file 1: Table S6).

DMR genes were also enriched in metabolic pathways, which are also relevant. In particular, one gene, *PKLR*, was aberrantly methylated in HCC; *PKLR* encodes a pyruvate kinase and is involved in the glycolysis process and important for cancer metabolism and tumor growth, [29,30]. In summary, these results suggest that abnormal DNA methylation and gene hydroxymethylation might be crucial participants in important cancer-related pathways in HCC.

### Transcriptional changes in key genes in cancer pathways are revealed by integration of multi-omics data

To reveal potential complications resulting from DNA modification changes on gene transcription, we further performed transcriptome profiling of the seven pairs of HCC samples and two HCC cell lines using RNA-Seq and Affymetrix microarray technologies. Based on the high-quality data, we performed pair-wise comparisons to identify differentially expressed genes (DEGs) in the seven pairs of HCC samples. Copy number variation (CNV) analyses were then used to address transcription changes due to gene CNVs (see the Methods section). If significantly upregulated genes were revealed through gene amplification in the HCC samples, we filtered out those DEGs. In addition, significantly downregulated genes resulting from gene deletion in the HCC samples were also removed. As a result, 5,848 upregulated and 1,586 downregulated DEGs in the HCC samples were identified (Additional file 1: Table S7). Furthermore, we examined the transcription level of these DEGs in the two HCC cell lines and found that most of these genes had a similar expression level as in the HCC samples but differed substantially from the non-HCC samples (Additional file 2: Figure S5).

Based on these data, we then examined key genes in the three cancer-related pathways that were enriched in the DMR genes (focal adhesion, *Wnt*, and *MAPK*). Notably, the *FAK* (*PTK2*), β-catenin (*CTNNB1*), and p38 MAPK (*MAPK12*) genes were all significantly upregulated in HCC. In particular, genes downstream of p38 MAPK including *MAPKAPK2*, *NFkB2*, *p53*, *CHOP* (*DDIT3*), *MSK* (*RPS6KA4*), *CREB* (*ATF4*), and *c-Myc*, were all upregulated, indicating activation of these signaling pathways in the HCC specimens (Figure 3).

### TSG candidate screening

To screen TSG candidates that might be aberrantly regulated by DNA methylation, we cross-matched the selected DEGs with genes that contained DMRs or DhMRs within 5 kb upstream of their TSS. As 5hmC is generated by TET proteins via oxidizing 5mC [15,16], we further required that hypermethylated genes should be hypohydroxymethylated and *vice versa*, hypomethylated genes should be hyperhydroxymethylated. As a result, expression levels of 124 DMR genes were negatively correlated with their methylation levels, while 22 DhMR genes were positively correlated between their expression and hydroxymethylation levels, in the three matched pairs. Majority (85) of the 124 DMR genes overlapped with the gene set identified in the five matched pairs. However, only 2 out of the 22 DhMR genes were replicated between the two comparison groups (Additional file 2: Figure S6). These results might indicate that DMRs are more stable than DhMRs in HCC populations. Based on these data and on reports in the literature, which indicated that the biological function of these genes is relevant to liver development or disease, we selected 18 candidate genes for further validation.

**Figure 4:**
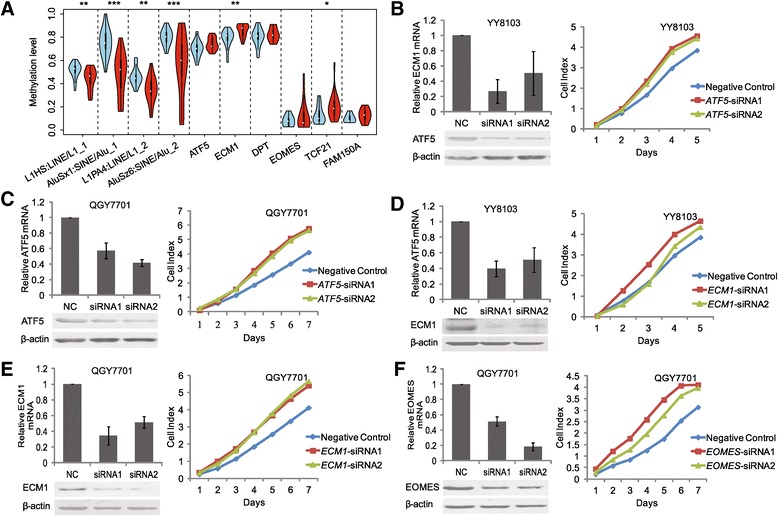
**The effect of siRNA knockdown on hepatocellular carcinoma cell growth. (A)** Validation of DNA methylation for biological replication (blue box indicate non-HCC and red box indicate HCC samples). Growth curve of YY8103 and QGY7701 cells treated with siRNA-NC, **(B, C)** *ATF5*-siRNA1 and *ATF5*-siRNA2, **(D, E)** *ECM1*-siRNA1 and *ECM1*-siRNA2, and **(F)** *EOMES*-siRNA1 and *EOMES*-siRNA2. The expression of genes was analyzed using both qRT-PCR and western blot.

Semi-quantitative RT-PCR was then used to detect the mRNA levels of the 18 genes in 20 additional pairs of HCC and non-HCC specimens (Primer sequences listed in Additional file 1: Table S8). Among the 18 genes, six genes including *FAM150A*, *TCF21*, *EOMES*, *ATF5*, *DPT*, and *ECM1* were frequently downregulated in 85%, 85%, 35%, 60%, 50%, and 60% of the 20 HCC samples, respectively, compared with non-HCC samples (Additional file 2: Figure S7). As all six of these genes only contained DMRs in their promoters, but not DhMRs, bisulfite sequencing can then efficiently validate DNA methylation changes for these genes. Therefore, we first performed bisulfite sequencing PCR (BSP) on three selected DMR genes (*FAM150A*, *TCF21*, and *EOMES*) in the three pairs of HCC samples for technical validation. The results confirmed significant differential methylation between HCC and non-HCC samples for each gene, indicating the accuracy of DMR detection by HMST-seq (Additional file 2: Figure S8, Additional file 1: Table S9). Based on these results, we further validated five selected DMR genes (*ATF5*, *ECM1*, *EOMES*, *FAM150A*, and *TCF21*) in 20 additional pairs of HCC and non-HCC samples for biological replication. A high-throughput illumina-sequencing-based BSP method that we developed recently was applied [31] (See Methods). Our results confirmed consistently higher methylation levels of all these genes in HCC samples in comparison with non-HCC samples (Figure 4A, Additional file 1: Table S9). In particular, *ECM1* and *TCF21* displayed significant differential methylation between HCC and non-HCC groups (*P* <0.05). Besides of key hypermethylated gene promoters, we also used this method to test four repetitive elements. Consistent with whole genome hypomethylation and hypohydroxymethylation tested by UPLC-MS/MS, the methylation levels of these repetitive elements tested by bisulfite sequencing were significantly lower in HCC samples (Figure 4A).

### Suppression of endogenous *ATF5*, *ECM1*, and *EOMES* promotes HCC cell line proliferation

**Figure 5:**
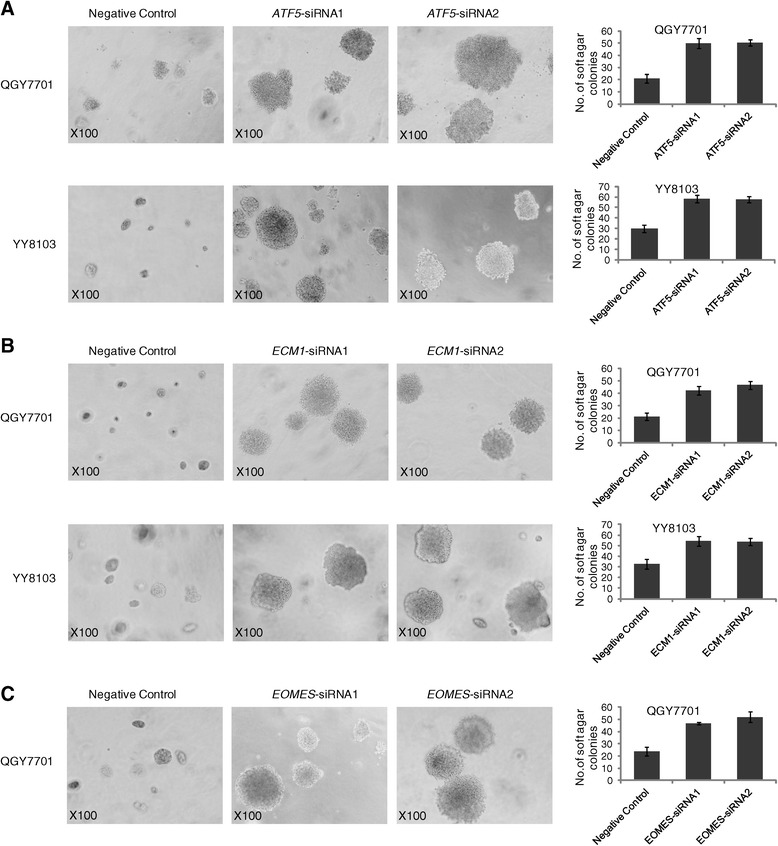
**siRNA knockdown affects anchorage-independent cell growth.** Anchorage-independent colony formation of YY8103 and/or QGY7701 cells treated with siRNA-NC, and **(A)** *ATF5*-siRNA1 and *ATF5*-siRNA2, **(B)** *ECM1*-siRNA1 and *ECM1*-siRNA2, **(C)** *EOMES*-siRNA1 and *EOMES*-siRNA2. The results showed that knockdown of the three genes including *ATF5*, *ECM1*, and *EOMES* can increase the Anchorage-independent colony formation. Original magnification is × 100.

To evaluate the potential contribution of the six target genes to HCC tumorigenesis, we next aimed to perform gene knockdown experiments in HCC cell lines. By evaluating the expression level of six target genes in available HCC cell lines using RT-PCR, we selected YY8103 and QGY7701 HCC cell lines, as in which *ATF5*, *EOMES*, and *ECM1* were all expressed. However, *FAM150A*, *TCF21*, and *DPT* were hardly expressed in almost all cell lines (Additional file 2: Figure S7). Based on these results, we designed two specific siRNAs against each gene of *ATF5*, *EOMES*, and *ECM1* (Additional file 1: Table S10) for knockdown experiments, using siRNA-NC as a negative control. Our data showed that the expression of these three endogenous target genes was efficiently knocked down in YY-8103 and QGY7701 cells by these siRNAs, as demonstrated through both real-time PCR assays and western blots (Figure 4B, C, D, E, and F). As a result, the transient transfection of siRNA1 and siRNA2 for these three genes enhanced the growth of YY8103 and QGY7701 HCC cells compared with cells transfected with siRNA-NC (*P* <0.05; Figure 4). Furthermore, analysis of colony formation in soft agar confirmed that among the four target genes, silencing *ATF5*, *ECM1*, and *EOMES* significantly promoted the anchorage-independent growth of YY-8103 and QGY7701 cells compared with the siRNA-NC control cells (*P* <0.05; Figure 5).

Taken together, these data support the hypothesis that knockdown of *ATF5*, *ECM1*, and *EOMES* consistently promoted the growth of existing HCC cells, suggesting that silencing these three genes may contribute to HCC oncogenesis and progression.

## Discussion

Previous studies have shown that hypermethylation of multiple TSGs contributes to HCC pathogenesis and represents a crucial event in HCC progression [6,9,11,32]. However, the recently discovered hydroxymethylation has brought an additional dimension of complexity to the methylation alterations observed in cancer. Most previous studies have used bisulfite treatment or methylation-sensitive enzyme digestion-based methods to investigate novel DNA methylation biomarkers, which cannot distinguish 5-hmC from 5-mC [12,13]. In addition to the heterogeneity of cancer samples or technological differences, inaccurate quantification of DNA methylation levels might also lead to inconsistent or even contradictory results between different studies [33]. Thus, distinguishing between 5hmC and 5mC assists in the identification of DNA methylation biomarkers for clinical and commercial use with better sensitivity and specificity.

Here, we applied a multi-omics strategy to vertically integrate epigenomic, genomic, and transcriptomic profiling to screen for novel epigenomic biomarkers and TSG candidates of HCC. We applied both the commonly used UPLC-MS/MS and the newly developed HMST-Seq technologies to simultaneously examine DNA methylation and hydroxymethylation levels. Globally, 5mC and 5hmC levels were reduced in HCC, as confirmed by UPLC-MS/MS. As HMST-Seq can only test CCGG *Msp*I sites, an increasing amount of 5mC was observed for CCGG sites, in contrast to whole genome 5mC tendency. This result not only reflects the limitation of HMST-Seq in detection of global 5mC pattern, but also indicates an uneven distribution of 5mC in the genome. On the other hand, as 5hmC were evenly distributed across the genome, consistently low levels of 5hmC across the genome-wide CCGG *Msp*I sites were also observed. Furthermore, DMRs and DhMRs rarely co-occurred at the same genomic region. These results might support the hypothesis that 5hmC is an intermediate of the demethylation process [15,16], and loss of hydroxymethylation might be also an epigenetic feature of HCCs [21].

We found that a significant number of DMR genes were enriched in several highly interrelated cancer signaling pathways, including the ‘Focal adhesion’, ‘Wnt’, and ‘MAPK’ pathways (Figure 2C). Furthermore, the activation of key signaling pathway genes, including *FAK* in ‘Focal adhesion’, β-catenin in ‘Wnt’, and *p38* and its downstream genes in ‘MAPK’, indicated the activation of these signaling pathways in HCC, which has been previously reported in HCC. Although we currently have no direct evidence that the methylation and hydroxymethylation changes in DMR and DhMR genes may directly contribute to the activation of these pathways, these two types of DNA modifications represent essential participants in heptocarcinogenesis. In addition to gene transcription repression, DNA modifications may also be important for alternative splicing [34], gene mutation [35], and chromatin remodeling [36] in tumorigenesis. Further studies are needed to examine these genes.

In addition to the genes found enriched by KEGG pathway analysis, we confirmed that the suppression of three novel genes by promoter hypermethylation can significantly enhance cell growth and progression in HCC cell lines, indicating their potential as TSG candidates. These three genes have never been reported in previous studies on aberrant DNA methylation of HCCs [6,9,11,32]. Of these three genes, *ECM1* has been reported to inhibit the activity of matrix metalloproteinase 9 (*MMP9*) through high-affinity protein/protein interactions [37]. Due to the significant downregulation of *ECM1* transcription, our data also support the significant upregulation of *MMP9* transcription in HCC, which agrees with previous observations of highly upregulated MMP expression in many solid tumors [38]. MMPs are key modulators of the tumor microenvironment, and it has been shown that MMPs not only regulate ECM turnover but also cell signaling pathways controlling cell growth, inflammation and angiogenesis in a non-proteolytic manner through various non-catalytic domains [38].

*ATF5* is a highly abundant liver-enriched transcription factor that belongs to the ATF/cAMP response element-binding family [39]. A study has indicated that transcriptional expression of *ATF5* is directly induced by *CHOP* and *ATF4*, which together with *ATF5* form the integrated stress response (ISR) pathway that is critical for alleviating damage accrued during acute stress [40]. Thus, the proapoptotic functions of *ATF5* and *CHOP* have been revealed [40]. In HCC, although *CHOP* was activated, *ATF5* was hypermethylated in its promoter and exhibited downregulated transcriptional expression. These results are in agreement with previous studies that confirmed downregulation of *ATF5* transcription [41] and protein expression levels [42] in HCC. Therefore, *ATF5* might be blocked in HCC, resulting in anti-apoptotic effects and cancer cell survival.

The T-box transcription factor eomesodermin (*EOMES*) has been found to have a regulatory role for CD8 T cell activity in humans [43] and plays a critical role in tumor immune surveillance and eradication [44]. The absence of *EOMES* in tumor-infiltrating lymphocytes correlates with enhanced lymph node metastasis in colorectal cancer [43].

## Conclusions

In summary, *ECM1*, *ATF5*, and *EOMES* may all serve as novel TSGs that are aberrantly hypermethylated at their promoters leading to their downregulated transcription, which results in HCC tumorigenesis and progression. In addition, we have identified three other genes (*FAM150A*, *TCF21*, and *DPT*) with promoter hypermethylation and significant transcriptional downregulation in HCCs. These six genes may comprise a gene panel that could be used for the clinical diagnosis and prognosis of HCCs with respect to promoter methylation and gene transcription levels.

## Materials and methods

### Sample materials

All HCC tissue specimens were obtained from patients who underwent surgical resection for their tumors and provided informed consent prior to liver surgery. The primary tumor specimens were immediately frozen at -80°C until DNA/RNA extraction. Specimens (approximately 1 cm^3^) of the tumor and adjacent liver tissue were collected from each patient, and the HCC diagnosis was confirmed through pathological examination. The HCC specimens used in this study were grouped according to differentiation grades II to III following the Edmondson-Steiner grading system. The clinical characteristics of the patients and tumors are summarized in Additional file 1: Table S1. This project and protocols involving human and animal tissues were approved by the ethics committee of the Research Ethics Committee of Shenzhen Third People’s Hospital (No. 2012-010), and conducted according to the Declaration of Helsinki. The use, for research purposes, of tumor tissues from clinically surgical removal was deemed exempt from a requirement for informed consent beyond the consent normally obtained for this clinical procedure.

Normal liver (including LO2 and WRL68) and HCC cell lines (including Hep3B, SK-hep1, Focus, Huh7, SMMC7721, MHCC97L, MHCC97H, MHCC-LM3, MHCC-LM6, PLC, HepG2, YY8103, QGY7701, QGY7703, BEL7402, BEL7404, and BEL7405) were also used in this study. Of these cell lines, Hep3B, SK-hep1, Focus, Huh7, SMMC7721, PLC, HepG2, YY8103, QGY7701, QGY7703, BEL7402, BEL7404, and BEL7405 were obtained from a commercial source (Institute of Chemistry and Cell Biology at Shanghai). In addition, the MHCC97L, MHCC97H, MHCC-LM3, and MHCC-LM6 cell lines were derived from published references [45,46] and were kindly provided by professor Yinkun Liu from the Liver Cancer Institute affiliated with Zhongshan Hospital in Shanghai.

### DNA and RNA extraction and cDNA synthesis

Genomic DNA was extracted from all samples using the DNeasy Tissue kit (Qiagen, Valencia, CA, USA), according to protocols recommended by the manufacturer. Total RNA was extracted from the HCC cell lines and frozen tissue samples, which were pulverized in liquid nitrogen using TRIzol reagent (Invitrogen, Carlsbad, CA, USA). To reduce the risk of genomic DNA contamination, 1 to 2 μg RNA was incubated with 2 U DNase I (Invitrogen, Carlsbad, CA, USA), 1 μL DNase buffer and, 0.4 μL RNase Out for 15 min at room temperature. The RNA concentration was determined by spectrophotometry, and the total RNA integrity was examined by visualization of the 28S and 18S ribosomal RNAs in a 1.2% agarose gel. First-strand cDNA was synthesized using the PrimeScript RT Reagent Kit (TaKaRa, Otsu, Japan) according to the manufacturer’s instructions.

### UPLC–MS/MS analysis

Genomic DNA (0.2 μg) extracted from all patients and cell lines was digested with 1 U DNase I, 2 U alkaline phosphatase, calf intestinal, and 0.005 U snake venom phosphodiesterase I at 37°C for 24 h. A microcon centrifugal filter device with a 3,000 D cutoff membrane was used to remove protein from the digested DNA samples by centrifuging at 12,000 rpm for 60 min. The mobile phase consisted of 0.1% formic acid (solvent A) and methanol containing 0.1% formic acid (solvent B). The flow rate was set to 500 μL/min. The enzymatically digested DNA samples (5 μL each) were injected for UPLC-MS/MS analysis with a LC gradient as follows: 0.0-4.0 min, 0 to 50% of solvent B; 4.0-6.0 min, 50% solvent B; 6.0-6.1 min, 50% to 5% of solvent B; and 6.1-15 min, 5% of solvent B. The separated analytes were detected using a 5500 Qtrap linear ion trap quadrupole mass spectrometer equipped with a Turbo V ion source operated in the ESI mode (AB Sciex) with Analyst software (Version 1.5). The Source and Gas were set as follows: gas 1, nitrogen (45 psi); gas 2, nitrogen (40 psi); ion spray voltage, 5,500 V; ion source temperature, 400°C; and curtain gas, nitrogen (30 psi). The mass spectrometer was operated in the multi-reaction monitoring mode (MRM).

### Data analysis for HSMT-Seq

Genomic DNA was extracted from all patients and cells using the QIAamp DNA Blood Mini Kit (Qiagen). HSMT-Seq library construction was performed following our previously reported protocol [22]. The libraries were sequenced using single-end 50 bp sequencing strategy with an Illumina HiSeq2000 sequencing system. The FASTQ sequence reads were mapped to a virtual human reference library (hg19) with no more than one mismatch after adapter removal and low-quality read filtering. The virtual library was constructed as follows. The human reference genome (hg19) was *in silico* digested with *Msp*I and *Nla*III, and we defined the DNA sequences between the nearest *Nla*III sites around each *Msp*I site in both directions as the virtual library reference for mapping. Unambiguous mapped tags were used for further analysis. The data for three libraries were normalized according to a previous method (Global Rank-invariant Set Normalization, GRSN) [47] based on the normalized tag counts, and the modification abundance of a specific CCGG site was determined as the ratio between the tag counts of two libraries. For instance, the hydroxymethylation level can be defined as the ratio between ‘C + mC + hmC’ and ‘C + mC’ tags. Furthermore, CCGG sites with significantly different tag counts (sequencing depth >10X, FDR <0.001) based on Poisson distribution with a ratio of tags between two libraries larger than 1 were determined to be significantly modified sites.

For a given genomic interval, the modification frequency of CCGG sites was defined as the ratio of significantly modified sites within all CCGG sites. Differentially methylated or hydroxymethylated regions (DMR or DhMR) between HCC and non-HCC samples were defined in the following steps: (1) The first five CCGG sites containing at least four CCGG sites with the same changing trend and a Wilcoxon rank-sum test *P* value <0.05 as a seed site for candidate DMRs was used; (2) a 3’ downstream adjacent CCGG with the same changing trend was then incorporated with this candidate DMR. Up to a 2,000-bp inter-distance was allowed between the two adjacent CCGGs, and a Wilcoxon rank-sum test was performed in the incorporated region; (3) Repeat these steps until the Wilcoxon rank-sum test *P* value is ≥0.05; and (4) the incorporated region was defined as a DMR or DhMR.

### Enrichment analysis

Functional enrichment analysis for genes with DMRs or DhMRs was performed using WebGestalt [48,49], which is freely accessible at [50].

### Data analysis for differentially expressed genes (DEGs)

The gene expression levels based on RNA-seq and Affymetrix microarray data were measured by the reads per kilobase of transcript per million reads (RPKM) and tags per million reads (TPM) algorithm, respectively. The differentially expressed genes (DEGs) were identified in the five pair-wise samples with Affymetrix microarray data by Wilcoxon rank-sum test and in each of the two pair-wise samples with RNA-seq data according to a previously published method [51]. Genes that were significantly expressed (FDR <0.05) in at least one group and regulated similarly in all the groups were further identified as differentially expressed genes (DEGs).

### Illumina-sequencing-based BSP

Illumina-sequencing-based BSP was performed based on a previously described strategy with minor adjustment [31]. PCR primers were designed using online software [52] and then concatenated 22 bp common sequences at their 5’ end, respectively, which would mediate high throughput sequencing linker extension during the following PCR procedure. All PCR products were about 500 bp. For the high throughput bisulfite-amplicon library construction using large scale of clinical samples, WaferGen Biosystem (WaferGenBiosystems) was employed. Briefly, 500 ng of genomic DNA extracted from whole blood were converted using EZ DNA Methylation-Gold Kit™ (ZYMO). For each sample, bisulfite converted genomic DNA, KAPA2G Robust HotStart ReadyMix (KAPA Biosystems), PCR primers, PCR primers mediated high throughput sequencing linkers with a barcode sequence were dispensed into one individual nanowell of a Smart MyDesign Chip, which contains 5,184 nanowells, by Smart Chip Multisample Nanodispenser. Thirty-six amplicons for each sample and 150 samples could be constructed as one library with barcode sequence classifying samples using one Smart MyDesign Chip. PCR amplification of Smart MyDesign Chip was conducted using the Techne Prime Thermal Cycler. PCR product was purified using the QIAquick Gel Extraction Kit (Qiagen). After analyzed by an Agilent 2100 Bioanalyzer (Agilent Technologies) and quantified by real-time PCR, the library was sequenced with pair end 250 bp using Illumina Miseq sequencer.

### Semi-quantitative RT-PCR

Reverse transcription (RT) was performed with 2 μg of total RNA treated with RNase-free DNase I. Semi-quantitative RT-PCR was then performed using primers for target genes (Additional file 1: Table S8). The length of the amplified fragments was 350 bp, and β-actin served as an internal reference. The primers for β-actin were as follows: 5′-TCACCCACACTGTGCCCATCTACGA-3′ (forward) and 5′-CAGCGGAACCGCTCATTGCCAATGG-3′ (reverse). The length of the β-actin amplicon was 295 bp. The PCR products were separated in 2% agarose gels containing ethidium bromide. The PCR reactions were performed in a volume of 20 μL using the TaKaRa PCR Kit. The reactions were performed at 94°C for 5 min followed by 30 to 35 cycles (for 18 target genes) or 25 cycles (for β-actin) at 94°C for 30 s, 55°C for 30 s, and 72°C for 30 s with a final extension at 70°C for 5 min. The PCR products were brought to 4°C at the end of the reaction, and they were then separated in 2% agarose gels containing ethidium bromide.

### Western blot

Cells were collected using a 2× loading lysis buffer (2× concentrations: 50 mmol/L Tris-HCl, pH 6.8, 2% sodium dodecyl sulfate, 10% 2-mercaptoethanol, 10% glycerol, and 0.002% bromophenol blue). Total protein extracts from cultured cells were subjected to protein gel electrophoresis using 12% SDS-PAGE, transferred to a Hybrid-PVDA membrane (Amersham Life Sciences) via a semi-dry electrophoretic transfer method, and treated with 20% methanol in a Tris-glycine buffer (25 mM Tris-HCl, pH 8.0, 0.2 M glycine, 0.1% SDS). After blocking with PBS containing 5% BSA, the membrane was incubated for immunoblotting analysis with a rabbit anti-myc polyclonal antibody (1:100; made in our laboratory) or anti-myc antibody (1:1,000) at room temperature for 2 h, followed by incubation with an IRDye 800DX-conjugated, affinity-purified goat anti-rabbit secondary antibody (1:1,000; Rockland). Signals were detected using the Odyssey Infrared Imaging System (LI-COR Biosciences). β-actin was used as a loading control.

### Identification of CNVs

Array-based comparative genomic hybridization (aCGH) was performed using Agilent 244-k arrays as previously described [23]. CNVs that were present in the matching normal sample were filtered, and the remaining CNVs were regarded as somatic variants.

### RNA interference

Three siRNAs directed against five target genes were designed on the Whitehead Institute Web Server (http://jura.wi.mit.edu/bioc/siRNAext/) and chemically synthesized (Shanghai GenePharma Co.) to target the different coding regions of each gene. The siRNA sequences are shown in Additional file 1: Table S6. In addition, siRNA-NC (5′-GAGUUAAAGUCAAAGUGACTT-3′ and 5′-GUCACUUUGACUUUAACUCTT-3′) was also synthesized. The siRNAs were transfected into the HCC cell lines, and cell growth was monitored. For siRNA transfection, 3 × 10^3^ HCC cells per well were seeded into 96-well plates. When the cells reached 30% to 50% confluence, they were transfected with synthetic siRNAs at a final concentration of 50 nM using the Lipofectamine 2000 Transfection Reagent (Invitrogen) according to the manufacturer’s instructions.

### Cell proliferation analysis

The cells were cultured for 7 days, and cell viability was measured using the ACEA RTCA kit (ACEA Biosciences, San Diego, CA, USA). A microelectronic cell sensor system was used to confirm the number of living cells. NSCLC cells (1 × 10^4^) were seeded into each sensor-containing well (19.6-mm^2^ surface with 150 mL of medium) of the microtiter plates. The electronic sensors provided a continuous (every 6 h), quantitative measurement of the cell index (reflecting the surface area covered by the cells) in each well. Cell growth was measured every 6 h for 96 to 144 h, and cell indexes were recorded for each well at all time points to assess cell viability. All experiments were independently repeated at least three times.

### Soft agar colony formation

For soft agar colony formation assays in 24-well plates, 2 × 10^4^ cells were plated and grown on plates containing 1% base agar and 0.5% top agar. The plated cells were incubated at 37°C for 21 days. The plates were stained with 0.005% crystal violet for 1 h, and colonies were counted under a dissecting microscope. All experiments were independently repeated at least three times.

### Data availability

The sequencing and processed data have been deposited in the NCBI SRA and GEO database under the accession number SRA092219 and GSE54141. All the UPLC-MS/MS raw data were deposited in figshare database (http://dx.doi.org/10.6084/m9.figshare.1117723).

## Additional files

Additional file 1:
**All supplemental tables (Tables S1 to S10) and corresponding captions.**
Additional file 2:
**All supplemental figures (Figures S1 to S8) and corresponding legends.**

## Abbreviations

5fC: 5-formylcytosine
5hmC: 5-hydroxymethylcytosine
5mC: 5-methylcytosine
CNV: Copy number variation
DEGs: Differentially expressed genes
DhMRs: Differentially hydroxymethylated regions
DMRs: Differentially methylated regions
HCC: Hepatocellular carcinoma
HMST-seq: Hydroxymethylation and methylation Sensitive Tag sequencing
non-HCC: Non-cancerous liver tissues
TSG: Tumor suppressor gene
TSS: Transcriptional start sites
UPLC-MS/MS: Ultra-performance liquid chromatography/tandem mass spectrometry
