# Supplementary material for: Integrated analyses of DNA methylation and hydroxymethylation reveal tumor suppressive roles of ECM1, ATF5, and EOMES in human hepatocellular carcinoma
Source: Genome Biol. 2014 Dec 3;15(12):533. doi: 10.1186/s13059-014-0533-9 (PMC4253612; doi:10.1186/s13059-014-0533-9)
Supplement: Additional file 2: — All supplemental figures (Figures S1 to S8) and corresponding legends. [file 13059_2014_533_MOESM2_ESM.pdf]

Supplementary figures

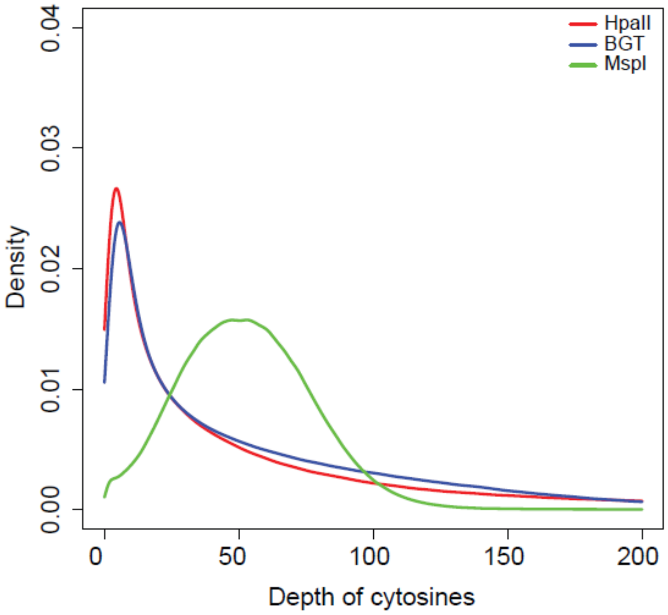

Supplementary figure S1 | Tag distribution of three libraries of N083062. Similar distributions of "C+mC" tags and "C" tags was presented, which indicated potential insufficient MspI digestion on glucocylated genomic DNA.

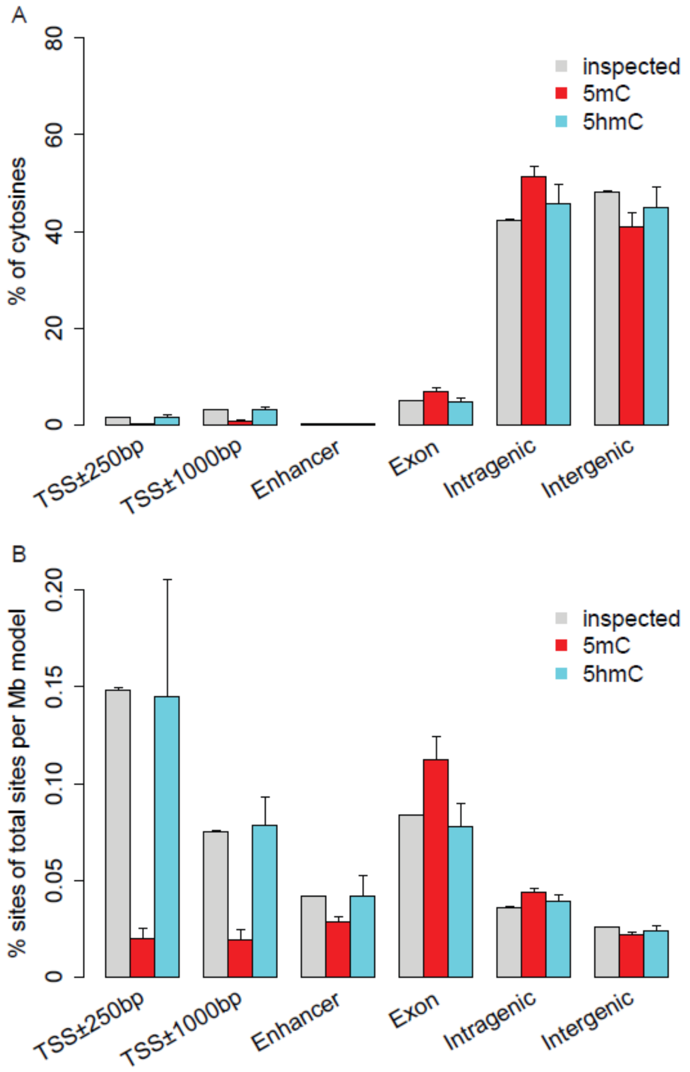

Supplementary figure S2| Global distribution of 5mC and 5hmC. (A) Percentage of 5mC and 5hmC within genomic regions. Genic features were extracted from the UCSC hg19 database and enhancer were downloaded from VISTA Enhancer Browser database. Each 5mC or 5hmC site is counted once: the cytosines located in previous regions are excluded from left to right. (B) Relative enrichment of modified sites within genomic regions. The observed percentage of 5mC (red) or 5hmC (blue) counts within these genomic regions out of all modified CCGG sites in human genome and the inspected percentage of CCGG counts (gray) within these genomic regions out of all CCGG counts in human genome are presented. All values of counts were normalized to the length of each region (per Mb).

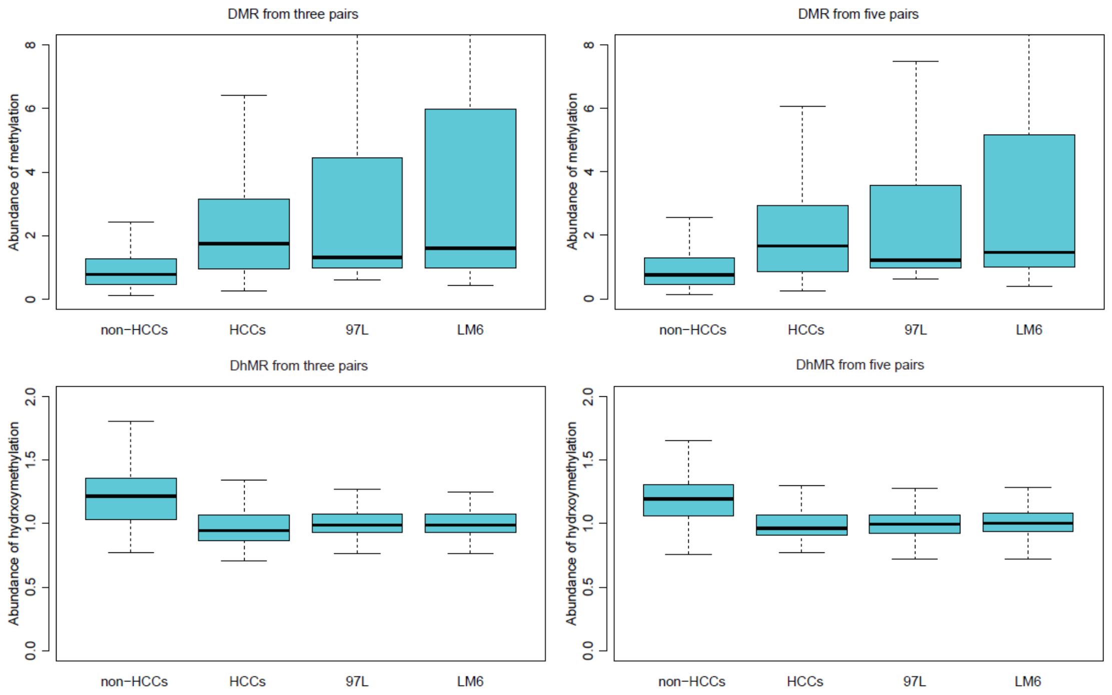

Supplementary figure S3 |Methylation and hydroxymethylation level of DMRs and DhMRs among HCC, non-HCC and two hepatocellular carcinoma cell lines.

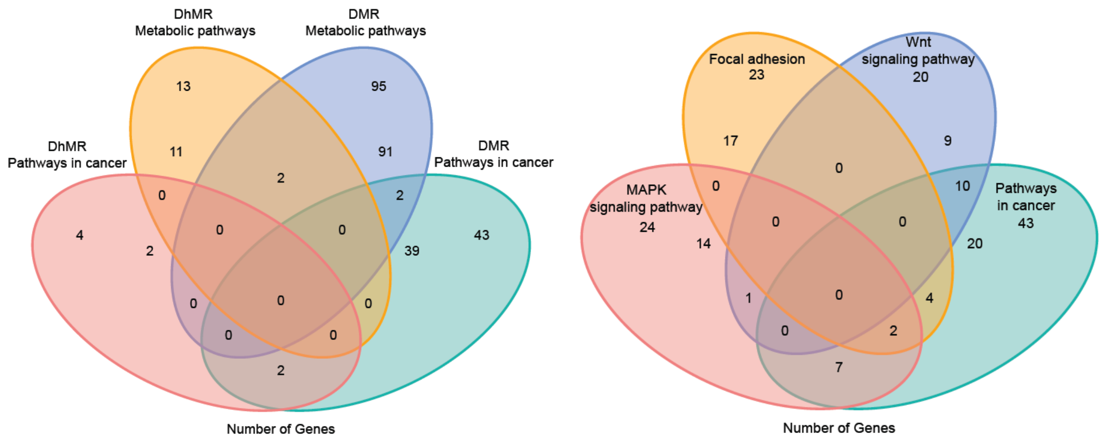

Supplementary figure S4 |Overlap of genes in enriched pathways. Overlap of genes with DMRs or DhMRs in “metabolic pathways” and “pathways in cancer”, and overlap of genes with DMRs in cancer related KEGG pathways.

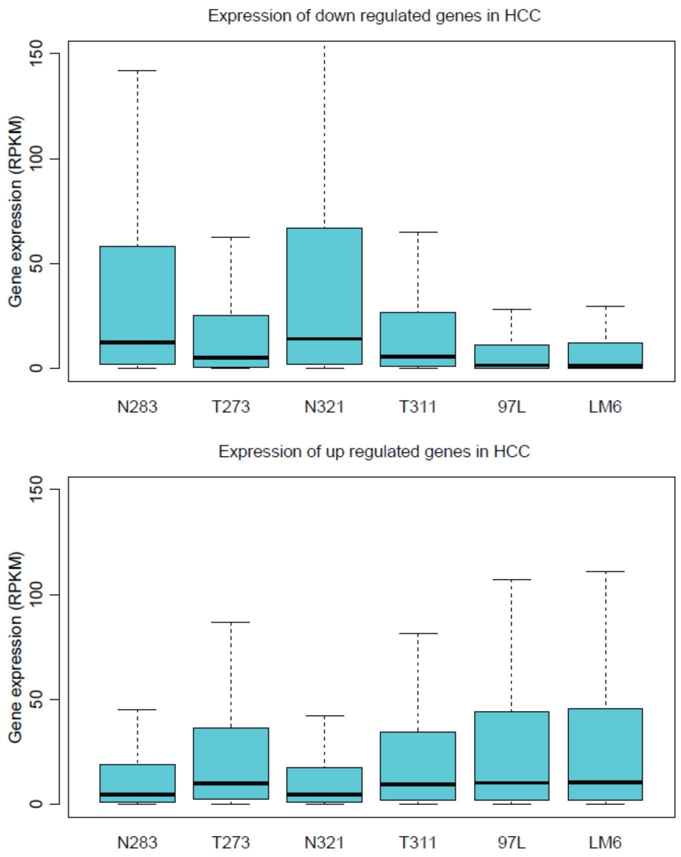

Supplementary figure S5 |Gene expression level of differentially expressed genes HCC, non-HCC and two hepatocellular carcinoma cell lines. N283 and N321 are non-HCC specimens, T273 and T311 are HCC specimens, and 97 and LM6 are hepatocellular carcinoma cell lines.

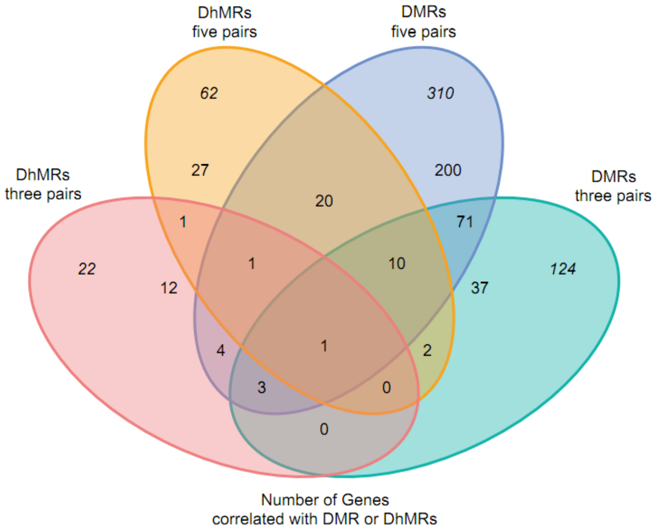

Supplementary figure S6 |Overlap of genes correlated with DMRs or DhMRs. Overlap of genes that negatively correlated with DMRs or positively correlated with DhMRs, where an opposite changing tendency between 5hmC and 5mC was observed, in three pairs and five pairs of HCC samples.

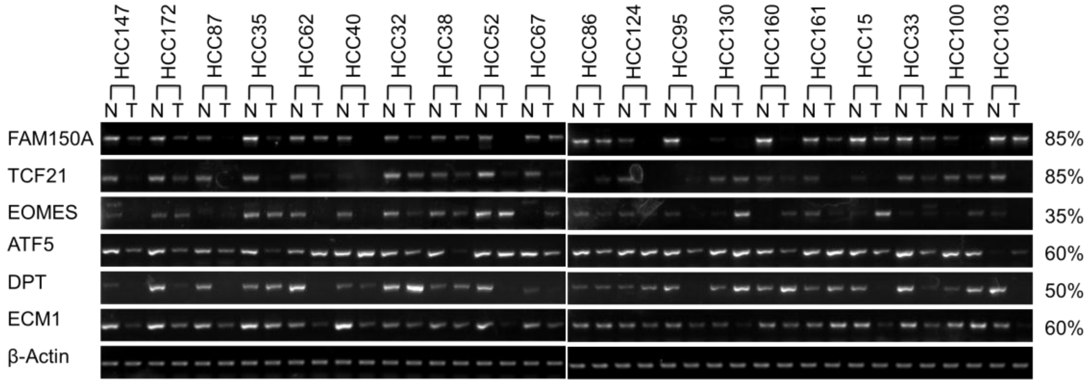

Supplementary figure S7 | Expression of six candidate genes in twenty pairs of HCC and non-HCC specimens. N represents non-HCC specimens, while C represents HCC specimens.

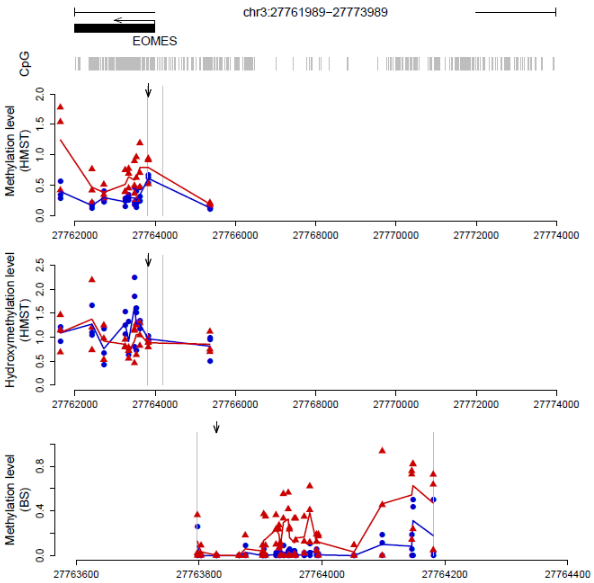

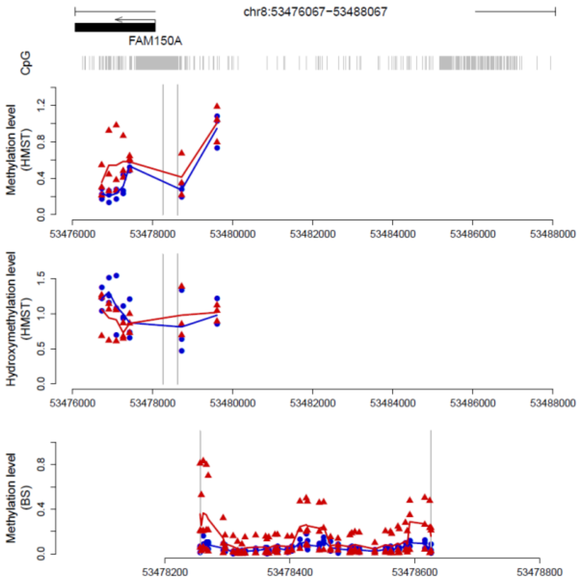

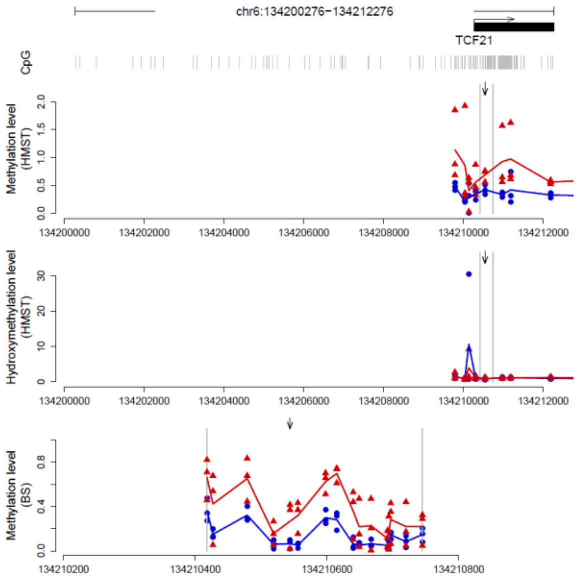

Supplementary figure S8 | Validation of three DMRs by bisulfite sequencing. The gene related to DMR and CpG sites are indicated at the top, followed by the methylation, hydroxymethylation level of DMRs detected by HMST techology and methylation level of validated region in DMR by bisulfite sequencing (BS) of HCC (red Triangle) and non-HCC (blue dot). The region between gray lines represents the validated region and the black arrow indicates the CpG sites in the validated region.

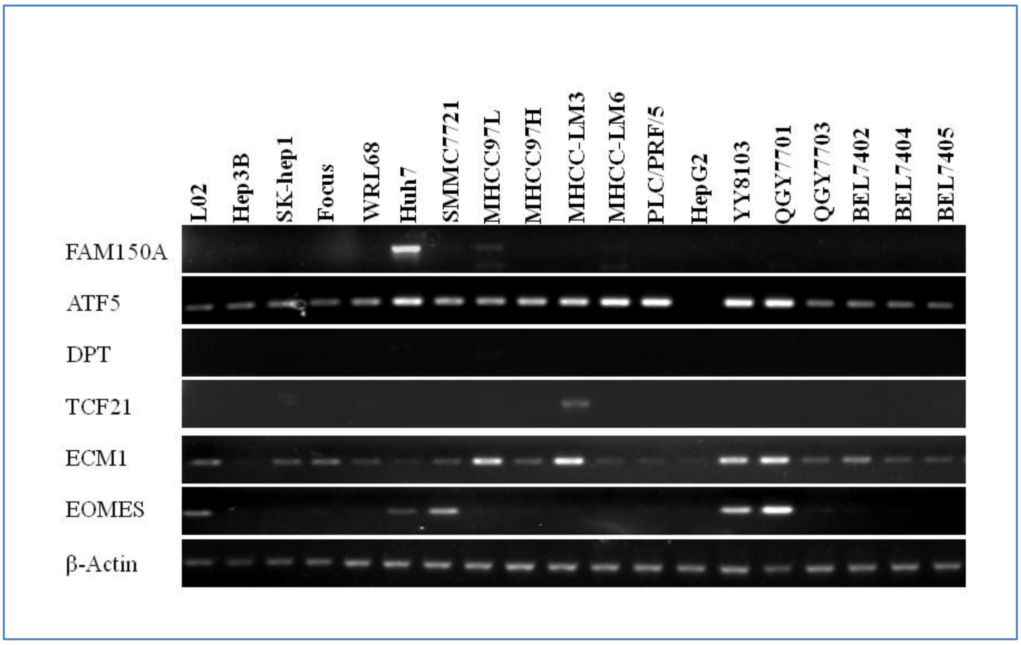

Supplementary figure S9. | mRNA expression pattern of six genes in HCC cell lines. Results of the semi-quantitative RT-PCR analysis of six genes in 19 liver cancer cell lines. β-Actin was used as an internal control. All PCR products were visualized by electrophoresis on 2% agarose gels.
